# P-450. Neonatal macrophages have an altered immunometabolic response to Mycobacterium tuberculosis which is modified by IFN-γ, IL-4 or lactate

**DOI:** 10.1093/ofid/ofaf695.665

**Published:** 2026-01-11

**Authors:** Cilian O Maoldomhnaigh, Donal Cox, Joseph Keane, Sharee Basdeo

**Affiliations:** Children's Health Ireland / Trinity Translational Medicine Institute, Dublin, Dublin, Ireland; Trinity Translational Medicine Institute, Dublin, Dublin, Ireland; Trinity Translational Medicine Institute, Dublin, Dublin, Ireland; Trinity Translational Medicine Institute, Dublin, Dublin, Ireland

## Abstract

**Background:**

Tuberculosis (TB) is the biggest infectious killer in the world and the most vulnerable time to infection is the newborn period. *Mycobacterium tuberculosis* (Mtb), the bacteria that causes TB, is phagocytosed by macrophages and subsequent metabolic responses are required in order to mount an appropriate immune response. This increase in glycolysis and decrease in oxidative phosphorylation (OXPHOS) is called the Warburg effect and we have previously shown that adult monocyte derived macrophages (MDM) undergo Warburg but that umbilical cord MDM (UCMDM) have an altered immunometabolic response, failing to reduce OXPHOS and producing less TNF following Mtb infection (Figure 1). We examined the effects of IFN-γ, IL-4 or lactate on MDM and UCMDM immunometabolic phenotype and function.

**Figure:**
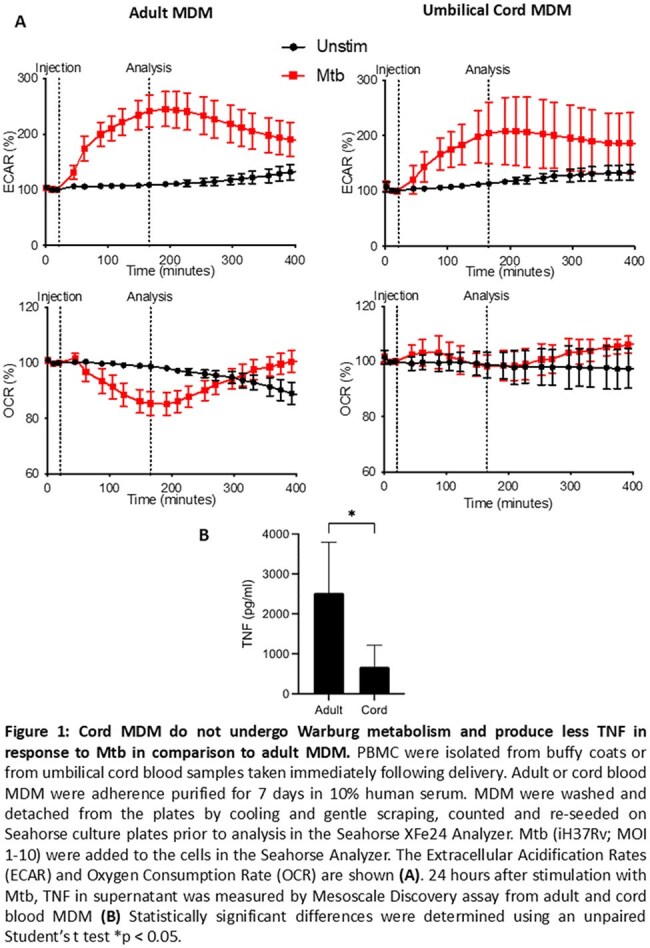

**Figure:**
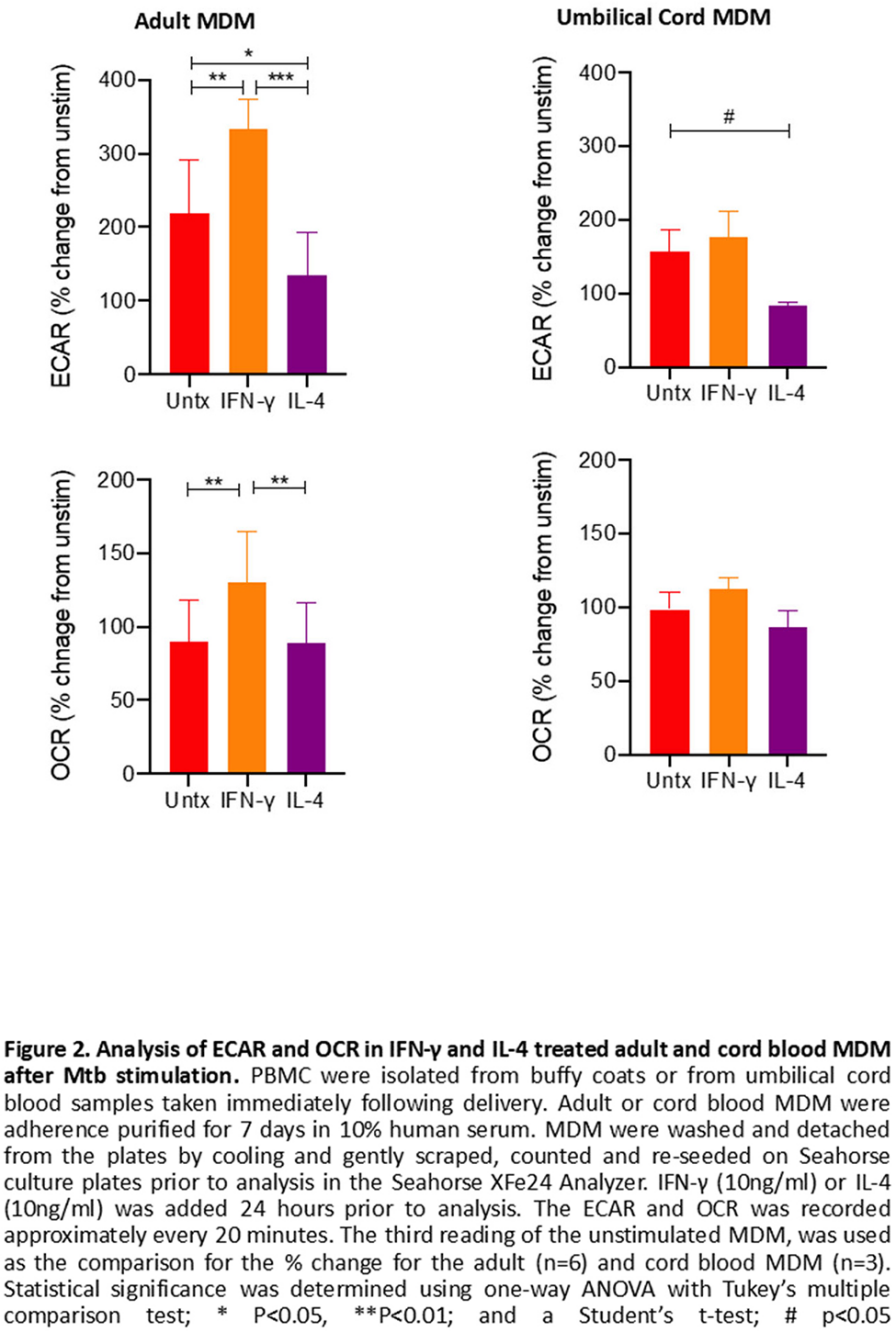

**Methods:**

MDM were derived from healthy adult buffy coats or from umbilical cord blood prior to analysis in the XFe Seahorse analyzer which measures the Extracellular Acidification Rate (ECAR) and the Oxygen Consumption Rate (OCR), surrogates of gycolysis and OXPHOS respectively . TNF and IL-1β in supernatant was measured by Mesoscale Discovery assay.

**Figure:**
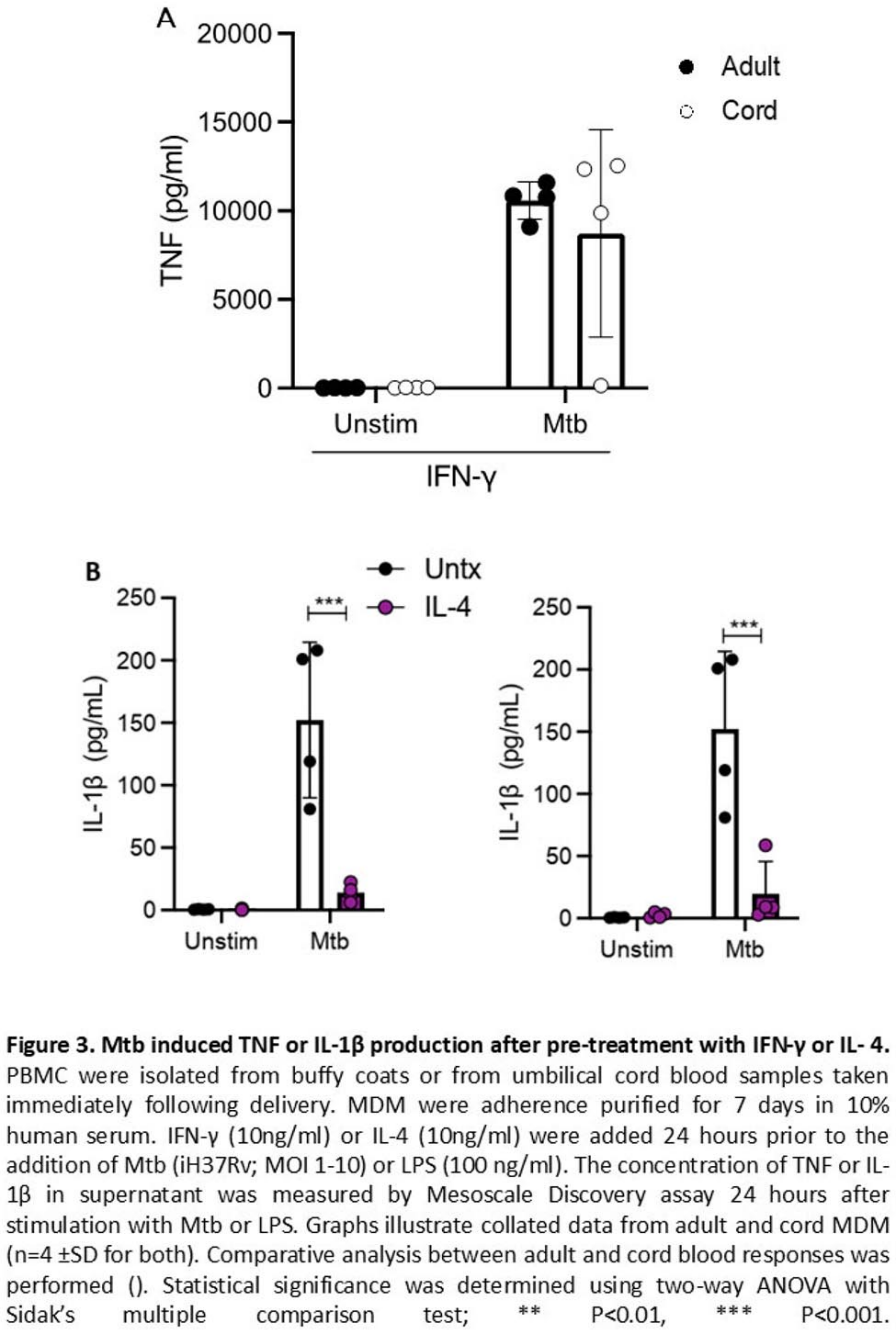

**Figure:**
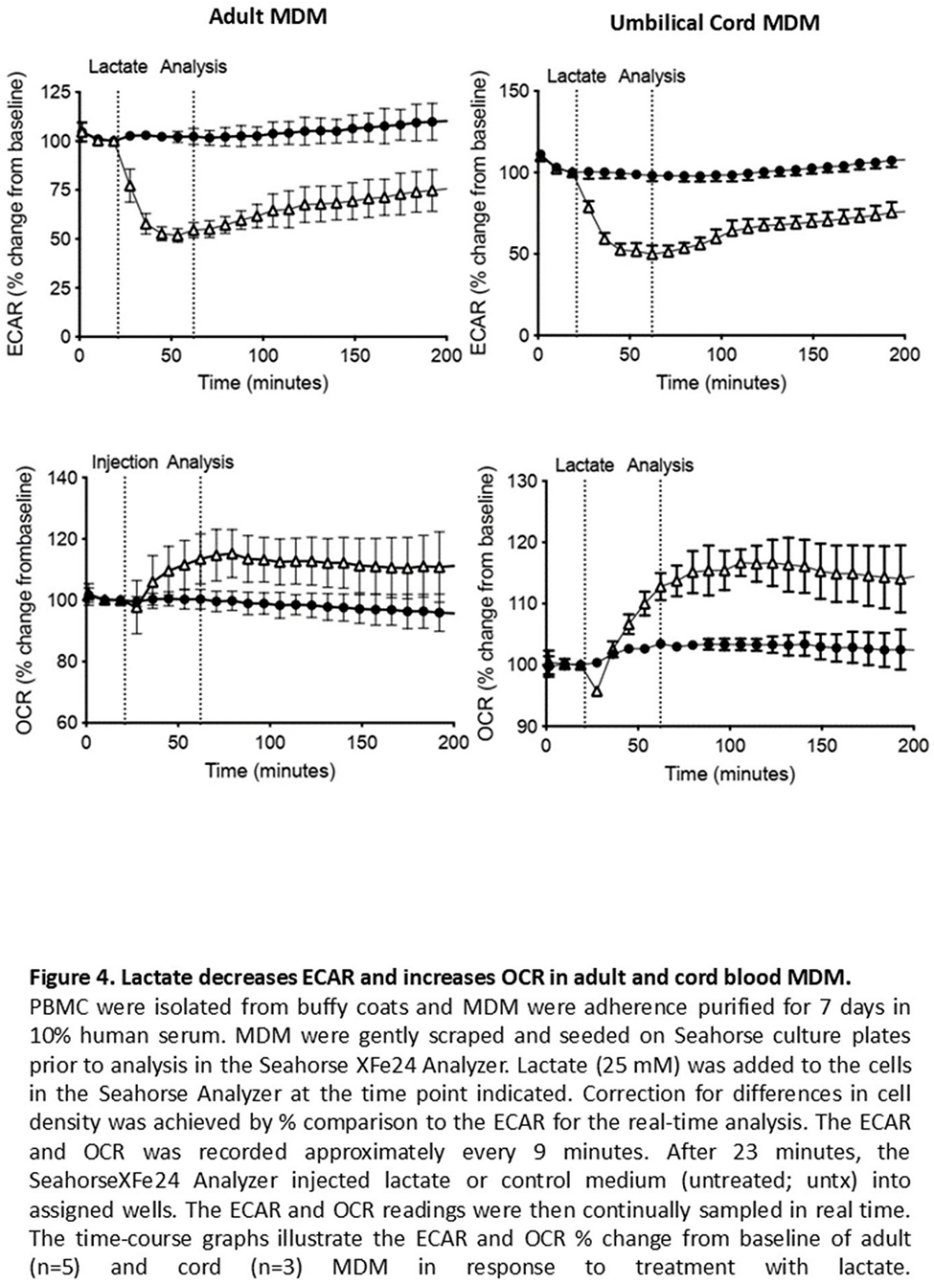

**Results:**

In MDM, IFN-γ increased and IL-4 decreased glycolysis and OXPHOS (Figure 2A). However in UCMDM, IFN-γ did not increase glycolysis or OXPHOS, while IL-4 decreased glycolysis but had no impact on OXPHOS (Figure 2B). Despite the lack of impact on metabolic function, IFN-γ increased UCMDM TNF production in response to Mtb, increasing to adult MDM levels (Figure 3A). IL-4 decreased IL-1β production in both MDM and UCMDM following Mtb (Figure 3B).

A consequence of increased glycolysis is an increase in extracellular lactate. The addition of exogenous lactate was found to have an immediate effect on metabolism, causing a decrease in glycolysis and an increase in OXPHOS in both MDM and UCMDM. (Figure 4).

**Conclusion:**

These data indicate that MDM and UCMDM exhibit distinct immunometabolic function upon stimulation which may underlie their differential ability to respond to infection and reveals potential avenues of immunomodulation which may inform therapeutic strategies for host-directed therapies for TB.

**Disclosures:**

All Authors: No reported disclosures

